# Vertically Aligned *n*-Type Silicon Nanowire Array as a Free-Standing Anode for Lithium-Ion Batteries

**DOI:** 10.3390/nano11113137

**Published:** 2021-11-20

**Authors:** Andika Pandu Nugroho, Naufal Hanif Hawari, Bagas Prakoso, Andam Deatama Refino, Nursidik Yulianto, Ferry Iskandar, Evvy Kartini, Erwin Peiner, Hutomo Suryo Wasisto, Afriyanti Sumboja

**Affiliations:** 1 Material Science and Engineering Research Group, Faculty of Mechanical and Aerospace, Institut Teknologi Bandung, Jl. Ganesha 10, Bandung 40132, Indonesia; 21andika@students.itb.ac.id (A.P.N.); 23720309@mahasiswa.itb.ac.id (N.H.H.); 2 National Battery Research Institute, Gedung EduCenter Lt. 2 Unit 22260 BSD City, South Tangerang 15331, Indonesia; evvy.kartini@n-bri.org; 3 Mekanisasi Perikanan, Politeknik Kelautan dan Perikanan Sorong, Jl. Kapitan Pattimura, Sorong 98411, Indonesia; bagas.prakoso@polikpsorong.ac.id; 4 Institute of Semiconductor Technology (IHT) and Laboratory for Emerging Nanometrology (LENA), Technische Universität Braunschweig, Hans-Sommer-Straße 66, 38106 Braunschweig, Germany; a.refino@tu-braunschweig.de (A.D.R.); n.yulianto@tu-braunschweig.de (N.Y.); e.peiner@tu-braunschweig.de (E.P.); h.wasisto@nanosense-id.com (H.S.W.); 5 Engineering Physics Program, Institut Teknologi Sumatera (ITERA), Jl. Terusan Ryacudu, Way Huwi, Lampung Selatan 35365, Indonesia; 6 Research Center for Physics, National Research and Innovation Agency (BRIN), Jl. Kawasan Puspiptek 441-442, South Tangerang 15314, Indonesia; 7 Department of Physics, Faculty of Mathematics and Natural Sciences, Institut Teknologi Bandung, Jl. Ganesha 10, Bandung 40132, Indonesia; ferry@fi.itb.ac.id; 8 Center for Science and Technology of Advanced Materials, National Nuclear Energy Agency (BATAN), South Tangerang 15314, Indonesia; 9 PT Nanosense Instrument Indonesia, Umbulharjo, Yogyakarta 55167, Indonesia

**Keywords:** silicon nanowire, nanowire array, silicon anode, *n*-type silicon anode, Li-ion battery

## Abstract

Due to its high theoretical specific capacity, a silicon anode is one of the candidates for realizing high energy density lithium-ion batteries (LIBs). However, problems related to bulk silicon (e.g., low intrinsic conductivity and massive volume expansion) limit the performance of silicon anodes. In this work, to improve the performance of silicon anodes, a vertically aligned *n*-type silicon nanowire array (*n*-SiNW) was fabricated using a well-controlled, top-down nano-machining technique by combining photolithography and inductively coupled plasma reactive ion etching (ICP-RIE) at a cryogenic temperature. The array of nanowires ~1 µm in diameter and with the aspect ratio of ~10 was successfully prepared from commercial *n*-type silicon wafer. The half-cell LIB with free-standing *n*-SiNW electrode exhibited an initial Coulombic efficiency of 91.1%, which was higher than the battery with a blank *n*-silicon wafer electrode (i.e., 67.5%). Upon 100 cycles of stability testing at 0.06 mA cm^−2^, the battery with the *n*-SiNW electrode retained 85.9% of its 0.50 mAh cm^−2^ capacity after the pre-lithiation step, whereas its counterpart, the blank *n*-silicon wafer electrode, only maintained 61.4% of 0.21 mAh cm^−2^ capacity. Furthermore, 76.7% capacity retention can be obtained at a current density of 0.2 mA cm^−2^, showing the potential of *n*-SiNW anodes for high current density applications. This work presents an alternative method for facile, high precision, and high throughput patterning on a wafer-scale to obtain a high aspect ratio *n*-SiNW, and its application in LIBs.

## 1. Introduction

Lithium-ion batteries (LIBs) are among the most robust energy storage devices due to their good cycle life, low self-discharge, and high energy density [1,2]. Those superiorities drive the utilization of LIBs in many applications, such as portable electronic devices, electric vehicles (EVs), and stationary energy storage [3]. However, traditional LIBs use graphite anodes, which possess a relatively low specific capacity (372 mAh g^−1^), limiting their achievable energy density [4]. Various materials with higher theoretical capacities have been pursued as alternatives for the graphite anode, such as Co_3_O_4_ (890 mAh g^−1^), Sn (994 mAh g^−1^), Ge (1625 mAh g^−1^), MgH_2_ (2038 mAh g^−1^), and Si (4200 mAh g^−1^) [5,6,7]. In particular, silicon has been introduced as the anode material for high energy density LIBs due to its large storage capacity, abundance, environmental friendliness, and suitable discharge voltage [8]. However, its significant volume expansion (up to 300–400%) during alloying with lithium leads to pulverization of active material and loss of electrical contact with the current collector, which restricts the performance of Si anode in LIBs, consequently [9].

Various strategies have been performed to improve the Si anode performance, e.g., introducing a dopant to enhance the conductivity, and nano-structuring to alleviate the volume expansion by providing buffer space around its structure [10,11,12]. In general, compared to undoped and *p*-type Si, *n*-type Si is favorable, as it can provide high conductivity derived from the high electron mobility in the silicon [13,14]. Furthermore, among various types of Si nanostructures, an array of one-dimensional (1D) Si nanowires (NWs) provides charge transport in one direction along the NW axis all the way to the current collector, enhancing the capacity and rate capability of the LIBs [15]. Proper spacing in the Si NW array enables facile strain relaxation in the NWs, which accommodates the volume changes during alloying with Li and improves the cycling stability of the Si anode [16].

Both bottom-up and top-down fabrication approaches have been employed to manufacture 1D Si nanostructures [17,18,19]. Bottom-up approaches may utilize deposition and templating methods that often require complex instruments, intricate methods, or toxic precursors [20]. Top-down methods, which are typically performed by combining lithography and etching processes, offer a controllable and facile procedure for large-scale fabrication of nanostructured Si. Among the nanoscale lithography techniques (e.g., nanoimprint lithography, colloidal nanosphere lithography, and electron beam lithography), photolithography is considered the most established method [21,22,23,24]. It enables the formation of a large variety of patterns with relatively short processing time, high accuracy of structural transfer, and suitability for wafer-scale production [25]. The deposited pattern will serve as a mask in the subsequent etching step, in which the exposed Si is removed, and the pattern is transferred onto the Si wafer, resulting in the formation of vertical nanostructures on the Si substrate.

Among various etching methods, reactive ion etching (RIE) utilizes a radio frequency (RF) electromagnetic field to bombard the etch target with ions and radicals produced in a plasma. Inductively coupled plasma (ICP) is introduced to the RIE system to obtain an independent control of ion density [26]. Besides that, by combining photolithography and ICP-RIE, the production cost could be potentially reduced due to the high throughput of the patterning process with homogenous shape and size. Moreover, compared to the Bosch process, which suffers from scalloping effects on the etched structures, ICP-RIE conducted at cryogenic temperatures can produce vertical Si NW arrays with high aspect ratios and smooth sidewalls [27]. Although high-aspect-ratio Si nanowires have been successfully produced by photolithography and cryogenic ICP-RIE, their use in LIBs is rarely reported [28].

Furthermore, nanostructured Si anodes have mostly been prepared by producing a slurry consisting of active materials, conductive additives, and binders, which were then coated onto a current collector [29,30,31]. However, the use of binders may reduce the conductivity of the anode. The interfaces among the active materials, binder, and current collector may also serve as initial crack points, which can limit the capacity and cycling stability of the LIBs [32]. On the other hand, free-standing anodes do not require a binder, additives, or a current collector, potentially reducing the production cost and overall resistance of the LIBs [33]. Moreover, due to their robust structure, free-standing Si anodes may enhance the cycling stability of LIBs by providing strong mechanical support during repeated charge/discharge processes [34]. Nanostructured black Si anodes had been fabricated using plasma etching of an *n*-type Si substrate [35]. However, despite its small NW diameter (450 nm) and high aspect ratio (~22), the black Si showed low initial Coulombic efficiency (i.e., ~23.9%) and poor cycling stability when used in a LIB, which could be associated with its uneven surface morphology and too-dense structure.

In this work, we demonstrate the fabrication of a vertically aligned *n*-type Si nanowire array (*n*-SiNW) as a free-standing anode for LIBs by combining photolithography and cryogenic ICP-RIE. This fast and precise method can produce an NW array with smooth morphology, high areal density, and adjustable dimensions. The as-produced NWs possess a high aspect ratio and are structurally attached to the Si substrate, which also serves as the current collector. A half-cell LIB was fabricated with the *n*-SiNW electrode, and its performance was compared to a respective counterpart with a plain/blank *n*-Si wafer electrode in terms of initial Coulombic efficiency, charge and discharge capacity, cycling stability, and capacity retention at high current densities. This work provides an alternative large-scale method for obtaining free-standing Si anodes with homogeneous and easily adjustable shapes and sizes.

## 2. Materials and Methods

### 2.1. Top-Down Fabrication of n-SiNW

Si wafers were purchased from SIEGERT WAFER GmbH, Aachen, Germany. They are *n*-type Si wafers doped with phosphorus having a crystal orientation of <100>, a thickness of 525 ± 20 µm, and a resistivity of 5–10 Ω cm. The *n*-SiNWs were directly carved from Si substrate by photolithography and cryogenic ICP-RIE. The Si wafers were first cleaned with acetone and dried with nitrogen blow. The wafers were then exposed to hexamethyldisilazane (HMDS) vapor during heating at 115 °C. The photoresist was deposited on the Si wafers by spin coating a diluted mixture of AZ 5214 E: AZ EBR (1:1) purchased from Merck Performance Materials Germany GmbH, Darmstadt, Germany, at 3000 rpm for 35 s on the Si wafer substrate, followed by soft-baking at 110 °C for 50 s. A photomask was used to transfer circular patterns to the substrate by exposing the sample to UV light generated by a 210 W Hg lamp for 13 s. This photolithography process was carried out by employing an MJB4 mask aligner from SÜSS MicroTec SE, Garching, Germany. The pattern was realized by dipping the substrate into AZ 726 MIF developer obtained from Merck Performance Materials Germany GmbH, Darmstadt, Germany for 25 s.

Once the circular photoresist patterns had been created on the Si wafer, cryogenic ICP-RIE was conducted using a SI 500C plasma etcher from SENTECH Instruments GmbH, Berlin, Germany. The etching was conducted using several optimized parameters: an ICP power of 500 W, an RF power of 6 W corresponding to an RF bias of −12 V, a temperature of −95 °C, a pressure of 1.0 Pa, an etch time of 5 min, an O_2_ flow of 12 sccm, and an SF_6_ flow of 119 sccm. To ensure good thermal dissipation during cryogenic cooling, a thermally conductive oil was applied between the Si wafer and the substrate holder, which was removed using acetone after the etching had been completed. At the same time, the remaining photoresist mask was also stripped off by acetone. To fit its size to the package of a typical coin cell Li-ion battery (LIB), the *n*-SiNW-containing wafer was then diced into a 1 × 1 cm^2^ piece (mass ≈ 131 mg).

### 2.2. Structural and Electrochemical Characterizations

X-ray diffraction (XRD) analysis was conducted utilizing an X-ray diffractometer (Bruker D8 Advance, Billerica, United States of America) using Cu Kα radiation (λ = 1.54060 Å). The diffraction peaks were analysed using PANalytical Expert Highscore Plus software. A scanning electron microscope (SEM HITACHI SU3500, Tokyo, Japan) was used to investigate the morphologies of the Si samples. The half-cell LIBs were assembled by employing *n*-SiNW and blank *n*-Si wafer electrodes, both having a die area of 1 × 1 cm^2^. The Si samples were directly used as the electrode without using any binder, additives, or current collector. Other essential components, a Li metal counter electrode, a polypropylene separator (Cellgard^®^ 2400), and a 1 M lithium hexafluorophosphate (LiPF_6_) electrolyte with 10 %wt fluoroethylene carbonate (FEC) in a 1:1:1 volume mixture of ethylene carbonate, dimethyl carbonate, and diethyl carbonate (EC/DMC/DEC), were stacked in an appropriate sequence in a CR2032 coin shell. All components were purchased from Xiamen Tob New Energy Technology Co., Ltd., Xiamen, China, except the FEC, which was bought from Sigma Aldrich, Singapore.

The half-cell assembly was performed in a glove box (Kiyon, Seoul, Korea), in which O_2_ and H_2_O concentrations were maintained at less than 0.1 ppm. The fabricated half-cells were rested for 24 h prior to the test to ensure the electrolyte was fully impregnated in the separator. To prepare a surface electro-active region and enable stable cycling performance, all cells were then pre-lithiated for 10 h to 0.1 V at a current density of 0.06 mA cm^−2^. To determine the capacity and cycling performance, the prepared cells were subsequently tested in a battery analyzer (Neware Battery Testing System, Shenzen, China) using the galvanostatic charge–discharge method within the potential range of 0.15 V to 1.0 V, at 0.06 mA cm^−2^ for 100 cycles at room temperature. The capacities of the cells were calculated with respect to the electrode areas (1 cm^2^). The rate capabilities were evaluated by charging and discharging at various current densities. The electrochemical impedance spectroscopy measurements were measured with an electrochemical workstation (GAMRY, Warminster, UK) after the pre-lithiation of the half cells in the frequency range of 1 MHz to 0.01 Hz, with an amplitude of 5 mV at room temperature. The resistance values of the cells were obtained from fitting with the ZSimpWin software.

## 3. Results and Discussion

**Figure 1:**
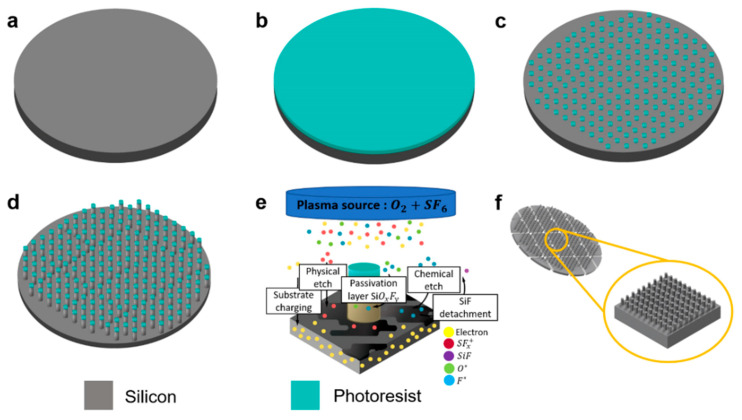
Schematic of the processing steps to fabricate the *n*-SiNW. (**a**) Preparation and cleaning of the Si wafer. (**b**) Spin coating of the photoresist on the wafer. (**c**) Circular photoresist pattern formation after photolithography. (**d**) ICP-RIE at cryogenic temperature resulting in an *n*-SiNW. (**e**) Mechanism of the etching process by ICP-RIE. (**f**) Photoresist removal and mechanical dicing to obtain a die area of 1 × 1 cm^2^.

Free-standing *n*-SiNW anodes were successfully fabricated by combining photolithography and cryogenic ICP-RIE (Figure 1). Photolithography was employed to create a photoresist mask pattern on a Si wafer. In the first step, the photoresist thin film was spin-coated and baked on a cleaned Si substrate (Figure 1a,b). Upon exposure to ultraviolet (UV) light, a circular pattern array was transferred to the photoresist. A developer solution was then used to selectively strip off the exposed photoresist area, leaving circular photoresist pattern arrays on the Si wafer (Figure 1c).

The cryogenic ICP-RIE enabled simultaneous passivation and etching processes (Figure 1d,e). In this process, a plasma discharge containing O_2_ and SF_6_ gases is generated inside a vacuum chamber to produce SF_x_ ions, and O and F radicals (O* and F*). The charged species (and the dragged radicals) are transported towards the Si substrate by an applied RF bias. A vertical bombardment of accelerated SF_x_ ions etch the Si wafer physically. Simultaneously, SiF species are desorbed due to chemical reactions between F* and the exposed Si atoms, performing chemical etching on the Si wafer. The etching removes Si from the areas of the non-masked surface, forming sidewalls around the masked area that subsequently turns into the vertical nanowire structure. At cryogenic temperatures, O* reacts with Si and F*, forming SiO_x_F_y_ that conformally adsorbs on the wafer surface and serves as a passivation layer against chemical etching. Due to the vertical direction of ion bombardment, a stable passivation layer can only be built up on the sidewalls, which protects the NWs from lateral etching. On the bottom surface between the NWs, the passivation layer is continuously removed by the physical etching of the impinging ions. At the end of the process, after exposing the etched substrate to room temperature, SiO_x_F_y_ becomes volatile, and the passivation layer on the NW sidewalls is removed. Cryogenic ICP-RIE enables the fabrication of Si NW arrays with various aspect ratios by adjusting the etching parameters (e.g., temperature, gas flow rate, ICP power, chamber pressure, and etching time) [27,36]. Besides, it can produce Si NWs with smooth sidewalls without a scalloping effect that may introduce stress concentration, resulting in the severe capacity fading of LIBs [37]. Finally, in order to fit the *n*-SiNW anode into a battery coin cell, the wafer was diced into 1 × 1 cm^2^ pieces (Figure 1f).

**Figure 2:**
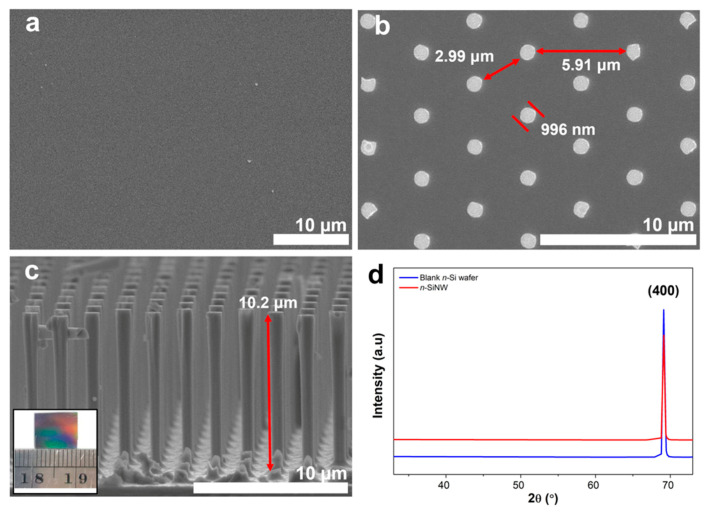
Top-down fabricated *n*-SiNW anode with an aspect ratio of ~10 compared to an unstructured n-Si substrate. Top view of SEM images for (**a**) plain/blank Si substrate and (**b**) n-SiNW. (**c**) The cross-sectional view of the free-standing *n*-SiNW anode (inset: the actual die dimensions of the *n*-SiNW anode). (**d**) X-ray diffraction (XRD) patterns of an *n*-SiNW and a plain/blank n-type Si wafer.

The nanowires obtained via photolithography and cryogenic ICP-RIE are homogeneous due to the high controllability of the process and the flexibility of the etching parameters [27]. The morphology of the obtained Si NW array was examined with a scanning electron microscope (SEM). It was first conducted on a blank *n*-type Si substrate (Figure 2a). The Si substrate has no specific morphology, except the flat surface, providing a perfect area for uniform deposition of the resist film. Starting from a corresponding surface, a homogenous nanowire array with predetermined spacing distance and diameter was obtained after etching (Figure 2b). The Si NWs had a diameter of ~996 nm and were ~3 μm from the closest wire. Side-view images further display that the nanowire was cylindrical with a height of ~10.2 μm, yielding an aspect ratio of ~10.2 (Figure 2c).

The obtained aspect ratio of *n*-SiNWs in this work is higher than that of a similar structure from another study with an aspect ratio of <7, which can be attributed to the selected etching parameters (e.g., gas flow rate and etch time) [38]. Vertical Si NWs with a high aspect ratio possess a high surface area with a large electrode–electrolyte interface, which can enhance electrolyte permeation and Li ion transport [39]. Furthermore, a well-ordered nanowire array is more desirable than a random array or disordered distributions of nanowires, which can lead to inefficient charge storage [40,41]. Moreover, the obtained *n*-SiNWs had direct attachment to the Si wafer, which strengthened the structural integrity of the anode. Figure 2d depicts X-ray diffraction (XRD) patterns of a plain/blank *n*-Si wafer and an as-fabricated *n*-SiNW array. The diffraction peak at 2θ = 69.13° for the plain/blank Si wafer and the *n*-SiNW corresponds to the <400> crystal orientation as the first reflection from <100>-Si [42]. An *n*-type Si with <100> orientation enables faster diffusion of Li ions, which is beneficial for reducing the volume expansion of Si anodes [43].

**Figure 3:**
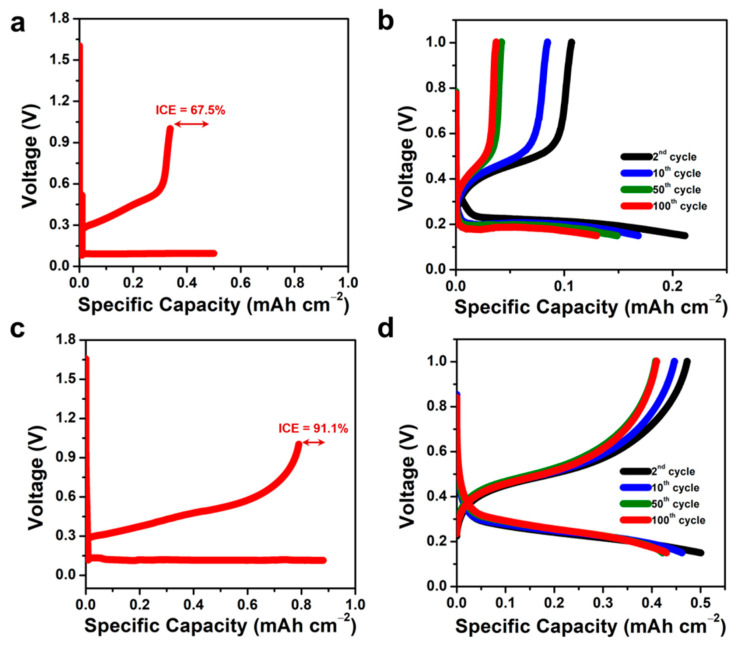
Galvanostatic discharge and charge profiles of the half-cell LIBs with plain/blank *n*-Si wafer and *n*-SiNW electrode. (**a**) Pre-lithiation cycle and (**b**) 2nd to 100th cycles of a LIB with blank *n*-Si wafer electrode. (**c**) Pre-lithiation cycle and (**d**) 2nd to 100th cycles of LIBs with *n*-SiNW electrode.

The electrochemical performance of plain/blank *n*-Si wafer and *n*-SiNW electrodes in half-cell LIBs were investigated by galvanostatic discharge–charge measurements (Figure 3). During the pre-lithiation cycle, the battery with a blank *n*-Si wafer electrode reached an initial Coulombic efficiency (ICE) of 67.5% with specific discharge and charge capacities of 0.50 mAh cm^−2^ and 0.34 mAh cm^−2^, respectively (Figure 3a). Noticeable capacity losses were observed in the following discharge–charge cycles, resulting in discharge capacities of 0.21 mAh cm^−2^, 0.15 mAh cm^−2^, and 0.13 mAh cm^−2^ at the 2nd, 50th, and 100th cycles, respectively (Figure 3b). The low discharge capacity of the LIB with the blank *n*-Si wafer electrode can be attributed to incomplete Li–Si alloying during the lithiation process [44]. The Li ions could not diffuse through the thickness of the blank Si wafer (~525 μm thick). Hence, only a small portion of the blank Si wafer could be alloyed with Li to form a Li–Si alloy, leading to the low discharge capacity of the LIB. Furthermore, during the cycling test, local volume expansion increased the internal stress of the Si electrode. That could produce cracks that would further consume active Li, generate new solid electrolyte interphase (SEI) layer, and degrade the LIB capacity, subsequently [45].

On the other hand, the half-cell LIB with *n*-SiNW electrode delivered improved specific discharge and charge capacities during the pre-lithiation cycle (0.88 and 0.80 mAh cm^−2^, respectively), generating an ICE as high as 91.1% (Figure 3c). The value of ICE increased to ~99% after three cycles and stabilized at the subsequent cycles. The battery with the *n*-SiNW electrode also showed improved discharge and charge capacities in the subsequent cycles—0.50, 0.42, and 0.43 mAh cm^−2^ at the 2nd, 50th, and 100th cycles, respectively (Figure 3d). In this case, lithiation in the vertical Si nanowires preferably took place in a radial orientation, providing a high surface area for the lithiation process [15,46]. Moreover, the small wire diameter of ~1 μm further shortens the diffusion path of Li ions. Therefore, a high proportion of Li ions can be effectively alloyed with Si, delivering a good discharge capacity for the LIB [15]. As the thickness of the remaining bulk Si substrate is much larger than the height of the *n*-SiNWs (i.e., ~50 times the *n*-SiNW height), the mass of the bulk substrate that does not contribute to the capacity is much larger than the NWs’ mass. Therefore, area-specific capacity was chosen as the more appropriate metric for comparison instead of gravimetric specific capacity. Besides that, area capacity measurements are typically performed to ensure compatibility between anode and cathode, as both electrodes face each other in battery cells [47]. Obviously, electrodes with high areal capacity are able to store more energy per unit area. Hence, it is a crucial parameter for the miniaturization of LIBs.

**Figure 4:**
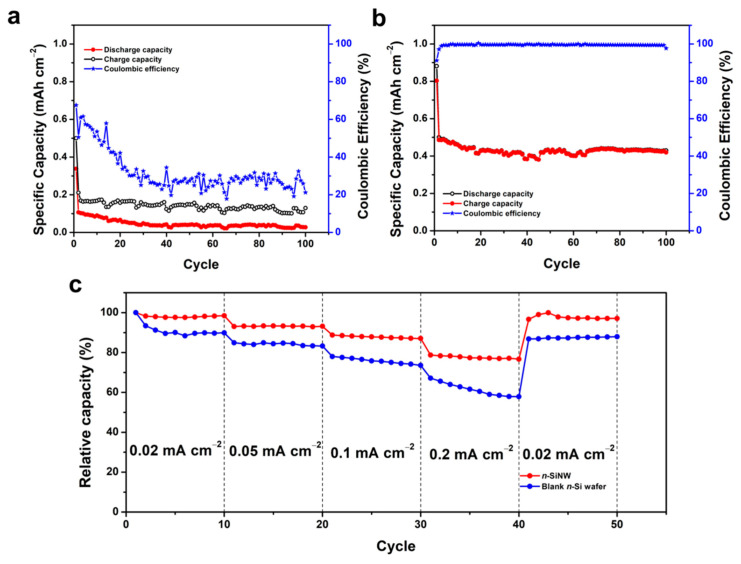
Electrochemical performances of LIBs with blank *n*-Si wafer or *n*-SiNW electrodes. Discharge–charge capacities and the corresponding Coulombic efficiencies at a current density of 0.06 mA cm^−2^ for 100 cycles of the batteries with (**a**) a blank *n*-Si wafer or (**b**) an *n*-SiNW electrode. (**c**) Capacity retention of LIBs with blank Si wafer and *n*-SiNW electrodes cycled at 0.02, 0.05, 0.1, and 0.2 mA cm^−2^.

The cycling performances of the half-cell LIBs with blank *n*-Si wafer and *n*-SiNW electrodes and their corresponding Coulombic efficiencies are given in Figure 4a,b. After 100 cycles of discharge–charge, the LIB with the blank Si wafer electrode (Figure 4a) exhibited low discharge and charge capacities (i.e., 0.13 and 0.03 mAh cm^−2^, respectively), and a low Coulombic efficiency (~21.2%). Furthermore, the Coulombic efficiency of the cell with a blank Si wafer was unstable throughout the cycling test, indicating the drawbacks of pristine and bulk Si as an electrode material for LIBs. The poor Coulombic efficiency of the battery could be associated with the continuous formation of an SEI, which severely consumes the active lithium in the LIB. Moreover, inhomogeneity of the formed SEI and a significant volume expansion of the blank Si wafer resulted in low capacity retention of the LIB (i.e., 61.4% after 100 cycles of the discharge–charge test). Bulk Si wafers may not be able to withstand the volume expansion, leading to plastic deformation and electrode failure upon delithiation [44].

The half-cell LIB with a *n*-SiNW electrode generated an average Coulombic efficiency of 99.5% during 100 cycles of discharge–charge at 0.06 mA cm^−2^ (Figure 4b). In addition, it also exhibited a relatively stable cycling performance, retaining 85.9% of its first discharge capacity after the pre-lithiation step. The free space between the nanowires can accommodate volume expansion, minimize internal stress, and keep the structural integrity of the electrode, resulting in high and stable Coulombic efficiency [8,48]. Moreover, the small diameter of the nanowires is able to mitigate the large volume change of the silicon, resulting in better cycling performance of the LIBs [49]. After 100 cycles, the cycling stability of the half-cell using an *n*-SiNW electrode was better than previous reports on Si NW anodes prepared by chemical vapor deposition (CVD) and metal-assisted chemical etching (MACE) that reported capacity retention of up to only 83% [19,50,51]. Additionally, the cycling stability of the cell with an *n*-SiNW electrode remained advantageous compared to some pristine Si nanoparticle and Si thin film anodes, which had less than 50% capacity retention after 100 cycles [52,53,54].

The rate capability tests of the LIBs with blank *n*-Si wafer and *n*-SiNW electrodes at various current densities are depicted in Figure 4c. At a high discharge rate of 0.2 mA cm^−2^, the LIB with a blank Si wafer electrode retained 57.9% of its discharge capacity at 0.02 mA cm^−2^. The cell also exhibited capacity retention of 87.9% when the current rate was reduced back to 0.02 mA cm^−2^. On the contrary, the capacity of the LIB with an *n*-SiNW electrode demonstrated capacity retention of 76.7% at 0.2 mA cm^−2^, and restored 97.1% of its capacity when the discharge rate was reduced back to 0.02 mA cm^−2^. In this case, the vertical arrangement of the nanowires provided a facile and fast Li ion diffusion pathway, resulting in the measured improved capacity retention at high current rates [15,43]. The good rate performance of LIBs with *n*-SiNW electrodes is also ascribed to the fast infiltration and circulation of the electrolyte in the nanowire array electrode, facilitating rapid ion transport during the electrochemical reactions [8]. Moreover, the high capacity retention after the current density was lowered back to 0.02 mA cm^−2^ indicates that the nanowire array is electrochemically stable and able to withstand high current charge–discharge rates.

**Figure 5:**
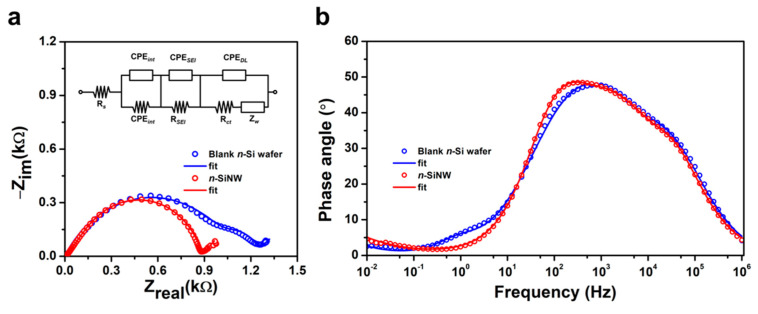
Electrochemical impedance spectroscopy (EIS) analysis of half-cell LIBs with blank *n*-Si wafer and *n*-SiNW electrodes after pre-lithiation cycles. (**a**) Nyquist plots of the cells with blank *n*-Si wafer and *n*-SiNW electrodes. The inset image is the equivalent circuit model used for fitting the EIS results. (**b**) Phase angle diagram of the cells with blank *n*-Si wafer and *n*-SiNW electrodes.

Electrochemical impedance spectroscopy (EIS) measurements for LIBs with blank *n*-Si wafer and *n*-SiNW electrodes were carried out after the prelithiation cycle at a voltage of 0.24 V. Figure 5a shows the results of EIS measurements and the corresponding equivalent circuit model [55]. *R_s_* is the Ohmic resistance of the whole cell, which can be expressed by an intercept at the high-frequency region [56]. The impedance of the imperfect contact due to the newly formed interface at the electrode is composed of the interface’s constant phase element (CPE*_int_*) and the interface’s resistance (R*_int_*) [55]. The impedance related to the characteristics of SEI is given by SEI’s constant phase element (CPE*_SEI_*) and the SEI’s resistance (R*_SEI_*). The last parallel components of the impedance consist of the capacitive nature of double-layer electrode/electrolyte interphase (CPE*_DL_*), charge transfer resistance at the electrode/electrolyte interface (R*_CT_*), and diffusion behavior of Li ions within the electrode which is expressed by the Warburg impedance (Z*_W_*) [57]. The fitted Nyquist plot is represented by a solid line, showing good agreement of the fitted parameters with the experimental results.

The similar *R*_S_ values (~10 Ω) for cells with blank *n*-Si wafer and *n*-SiNW electrodes can be attributed to the same half-cell systems that were used in the experiments. The cell with an *n*-SiNW electrode had a lower R*_INT_* (9.87 Ω) than its counterpart with a blank *n*-Si wafer electrode (14.66 Ω). These results suggest the small resistance associated with the newly formed interfaces due to the volume expansion in the *n*-SiNW, further affirming the benefit of the nanowire structure in mitigating the volume changes in the Si anode. The *R*_SEI_ value can be influenced by the type of electrolyte and the volume expansion of Si, which affect the growth of the SEI [58]. Since both cells used the same electrolyte, the lower *R*_SEI_ of the cell with the *n*-SiNW electrode (784.6 Ω) in comparison to the cell with the blank *n*-Si wafer (1057 Ω) can be attributed to the more controlled volume expansion of Si nanowires, which stabilizes the growth of the SEI during alloying with Li [15]. Furthermore, the smaller *R*_CT_ of the LIB with an *n*-SiNW electrode (74.5 Ω) than its counterpart with a blank *n*-Si wafer electrode (155 Ω) also suggests the structural benefit of the nanowire array electrode over its bulk structure. In particular, the nanowire array structure provides facile charge transport channels, leading to fast kinetics of charge displacement at the electrode–electrolyte interface [59]. Consequently, the *n*-SiNW electrode has improved kinetics during electrochemical alloying and dealloying, resulting in a higher capacity and enhanced rate capability.

The phase angle diagram for both cells is shown in Figure 5b. At the high to the middle-frequency region (1 MHz–1 kHz), the cells with blank *n*-Si wafer and *n*-SiNW electrodes showed similar responses, as the Ohmic resistance dominates the impedance response [60]. The reversal of the phase angle at 1 kHz–10 Hz is related to the impedance of SEI in both cells. The relatively high phase angle of the cell with *n*-SiNW suggests that the *n*-SiNW electrode produced a more stable SEI compared to the blank *n*-Si wafer, which is in agreement with a higher R*_SEI_* of the cell with the blank *n*-Si wafer than the cell with the *n*-SiNW electrode [61]. The charge transfer behavior of both cells in the phase angle diagram is characterized in the frequency range of 10 Hz–0.1 Hz. At this range, the lower phase angle of the cell with *n*-SiNW indicates that the *n*-SiNW electrode is able to mobilize a higher number of electrons at the electrode–electrolyte interface compared to the blank *n*-Si wafer electrode. These data are in line with the fitting results of Nyquist plots, where the cell with the *n*-SiNW electrode has a lower R*_CT_* than its counterpart with the blank *n*-Si wafer electrode.

## 4. Conclusions

Free-standing Li-ion battery (LIB) anodes made of *n*-SiNW were successfully realized through a combination of photolithography and cryogenic ICP-RIE. Homogeneous *n*-SiNWs with a predetermined diameter of ~996 nm and a high aspect ratio of ~10.2 were well “carved” on commercial *n*-type Si wafer substrates. A half-cell LIB with a free-standing *n*-SiNW electrode typically generated a promising ICE as high as 91.13%. A large areal capacity of 0.43 mAh cm^−2^ after 100 cycles of cycling test could be maintained by the designed structure, retaining 86% of its initial capacity after the pre-lithiation. Furthermore, it exhibited high capacity retention—up to 77% of its initial value at a current density of 0.2 mA cm^−2^. This promising performance is attributed to a facile and short-diffusion Li ion pathway, and fast infiltration and circulation of the electrolyte in the vertically aligned Si nanowire array anode. In the next development steps, further optimization can be expected by employing higher aspect ratio nanowires (of either larger height or smaller diameter) and combining *n*-SiNW with other active materials to form the composite electrode.

## Data Availability

Not applicable.
